# Effectiveness of topical corticosteroids in addition to antiviral therapy in the management of recurrent herpes labialis: a systematic review and meta-analysis

**DOI:** 10.1186/s12879-015-0824-0

**Published:** 2015-02-21

**Authors:** Nasira Arain, Sharath CV Paravastu, Mubashir A Arain

**Affiliations:** School of Health and Related Research, The University of Sheffield, Sheffield, UK; Faculty of Nursing, University of Calgary, 2500 University Drive NW, T2N 1 N4 Calgary, AB Canada

**Keywords:** Herpes labialis, Recurrent herpes infection, Cold sores, Corticosteroids and Herpes infection, Meta-analysis

## Abstract

**Background:**

Recurrent herpes labialis (RHL) is one of the most common viral infections worldwide. The available treatments have limited efficacy in preventing the recurrence of ulcerative lesions and reducing the duration of illness. The objective of this review was to identify the effectiveness of topical corticosteroids in addition to antiviral therapy in the treatment of RHL infection.

**Methods:**

A systematic review of randomized clinical trials comparing the efficacy of combined therapy (topical corticosteroids with antiviral) with placebo or antiviral alone in the management of RHL was conducted. MEDLINE, EMBASE, CINAHL, Web of Science, the Cochrane library, and Google Scholar databases were searched. We used RevMan software to conduct the meta-analysis. A fixed-effects model was used for mild to moderate heterogeneity, whereas a random-effects model was used for significant heterogeneity. Heterogeneity among trials was established using I^2^ and chi-square test for heterogeneity.

**Results:**

Four studies that fulfilled the selection criteria were included in this review. The total number of participants across included studies was 1,891 (range, 29 to 1,443). The antiviral drugs used were acyclovir, famciclovir, and valacyclovir. Corticosteroids used were 1% hydrocortisone and 0.05% fluocinonide. Pooled results showed that patients receiving combined therapy had a significantly lower recurrence rate of ulcerative lesions compared to those in both the placebo group (OR, 0.50; 95% CI, 0.39-0.66; P < .001) and the antiviral treatment alone group (OR, 0.73, 95% CI, 0.58–0.92; P = .007). The healing time was also significantly shorter in combined therapy in comparison to placebo (P < .001). However, there were no significant differences in healing time between combined therapy and antiviral alone. The adverse reactions in combined therapy were not significantly different than the placebo group (OR, 1.09; 95% C, 0.75-1.59; P = .85).

**Conclusion:**

Treatment with combined therapy is safe and more effective than placebo or antiviral alone for preventing the recurrence of ulcerative lesions in RHL infection.

## Background

Herpes labialis infection is a global public health problem, with 15 to 40% of the population who experience symptomatic outbreak [1]. Infection rates are high among HIV positive patients; about 95% of patients are seropositive to herpes simplex type I antigen [2]. In developed countries, one third of the population suffer from recurrent herpes labialis. (RHL) Between 20 to 40% of adults become infected with herpes simplex infection at some point during their lifetime [3]. Detectable serum antibodies against herpes simplex virus are more prevalent in lower socioeconomic groups [4]. Over the last 20 years, prevalence has increased globally and the prevention of RHL poses a big challenge for the 21^st^ century. The infection is difficult to eradicate and treatment has minimal impact on reduction and prevention of herpes infection. To date, no vaccination has been successful so far in humans to prevent the primary infection of RHL [5].

Herpes simplex labialis (HSL) is a contagious infection that appears as a rash of the skin, usually involving the lips but can affect oral membranes, and is characterized by blisters with pain and occasional itching [6]. There are different sequential stages of lesion including prodrome, redness, papule, vesicular, ulcer, hard crust, dry flaking, and normal skin (complete epithelisation). Ulcerative lesions take longer time to heal, which can adversely impact quality of life [7]. The diagnosis is typically based on clinical history and examination; in some cases, however, specific laboratory tests may be required [8]. The condition is usually mild, but some patients may have severe disease that may affect internal organs or lead to secondary bacterial infections [9].

Current treatment options for HSL include oral antiviral drugs, antiviral ointment or other topical applications (e.g., zinc oxide, zinc sulphate), and anesthetic creams for symptomatic improvement [10]. Treatment needs to be initiated promptly to achieve favorable results. Despite the above mentioned available treatment options, antivirals are commonly used and can slightly reduce the duration of herpes lesions by restraining the multiplication of the virus [11]. However, treatment with antiviral alone is not very effective and in most cases only has a minor effect on the duration of illness. Therefore, researchers have suggested adding corticosteroids to antiviral agents to increase the responsiveness of lesions [12,13] because herpes infection also triggers immune response. However, some controversy exists about the addition of corticosteroids, as steroidal contents may worsen the infection by reducing the natural defense system against the infection [14].

Several countries such as US, Germany, and Netherlands have approved antiviral with topical corticosteroids for the treatment of HSL [15]. However, it has not been licensed yet in other countries such as the UK. Therefore, it is important to understand the role of topical corticosteroid therapy plus anti-viral agent in the management of RHL infection. The purpose of this review was to determine the effectiveness of topical corticosteroids in addition to antiviral therapy (combined therapy) compared to antiviral therapy alone or placebo in the management of RHL infection using a systematic review and meta-analysis.

## Methods

### Search methods for identification of studies

We searched electronic databases including MEDLINE via ovid, EMBASE, CINAHL, Web of Science, and Cochrane Library. Searches were limited to English-language reports of human studies from 2000, which marked the first clinical trial conducted on topical corticosteroids and antiviral therapy on humans, to 2013.

A combination of medical subject headings (MeSH) and key word searches were used to retrieve the relevant literature in this review. All keywords were entered either with “OR” and “AND” boolean operators and were used with $/* where appropriate.

The following MeSH search terms were used:

“herpes simplex”; “herpes simplex virus”; “herpes labialis”; herpes labialis viruses”; “recurrences”; “recurrent herpes”; “topical administration”; “anti-infective agents”; “topical drug administration”; “corticosteroids”; “steroid”; “antiviral agents”; antiviral drugs”.

The following key words were used:

herpes simplex; herpes labialis; herpes virus; recurrent herpes labialis; prevention of herpes; treatment of herpes labialis; antiviral for herpes; antiviral + corticosteroid + herpes; topical antiviral for herpes; topical treatment + herpes; herpes labialis treatment + antiviral + corticosteroid.

Additional studies were sought through a review of the reference lists of obtained reports and other relevant reviews on the topic. Grey literature was also searched using Google Scholar and the ClinicalTrials.gov website (http://clinicaltrials.gov).

### Inclusion and exclusion criteria

Original research articles of randomized controlled clinical trials (RCTs) or controlled clinical trials comparing the effectiveness of topical corticosteriods in addition to antiviral (combined therapy) versus antiviral alone or placebo for the treatment of RHL were eligible for inclusion. Pilot clinical trial studies with similar interventions and outcomes were also included. We excluded observational studies and studies that evaluated the treatment for primary herpes labialis, as well as case reports, conference presentations, and editorials. Studies of healthy immuno-competent adolescents (12–17 years) and adults (≥18 years) with a history of RHL were included, irrespective of gender, socioeconomic status, and race. Two types of interventions were considered for the inclusion: (1) combined therapy versus antiviral therapy alone; or (2) combined therapy versus placebo. The primary outcomes of interest were the development of classical lesions and reduction in pain. Secondary outcomes were healing time, adverse events, and reduction in size of the lesions.

All authors were involved in determining inclusion.

### Data collection and analysis

Data collection and analysis was performed using Review Manager (RevMan Version 5.2, Copenhagen: The Nordic Cochrane Centre, the Cochrane Collaboration, 2012). Data from the studies were extracted using data extraction form. Risk of bias was assessed using the following key domains: randomization, allocation concealment, sample size, blinding (single, double or triple), and attrition rate [16].

Odd ratios (ORs) were used for dichotomous outcomes to measure the strength of association along with 95% confidence intervals (CI). P < .05 was considered significant. For continuous outcomes, mean differences between the groups along with 95% CIs were measured.

The meta-analysis of continuous data requires means with variance (or SD) to pool data. Outcomes of continuous variables were transformed from medians with interquartile ranges (IQR) to means with SD, using the following formulas [17]:

1. Conversion of median into mean: *χ* ≈ *a* + 2*m* + *b*/4
2. Conversion of range into variance: S = b-a/4 = R/4

[X = mean, a = smallest value, b = largest value, m = median, S = variance, R = range].

A fixed-effects model was used for mild to moderate heterogeneity, whereas random-effects model was used for those outcomes when the chi-square test for heterogeneity was significant (P < .10) and I^2^ was higher than 50%.

## Results

### Description of the selected studies

**Figure 1:**
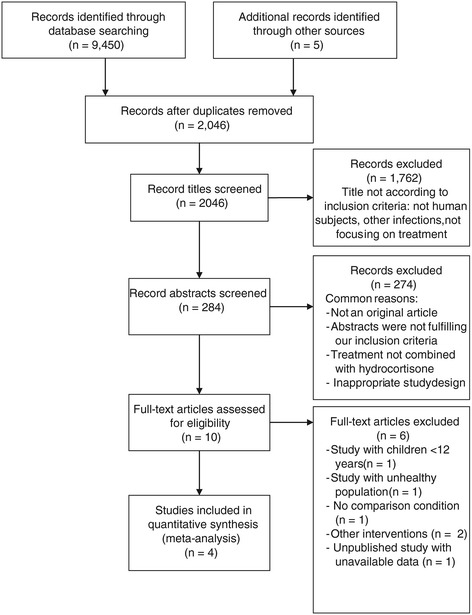
**PRISMA flow diagram.**

A total of 9,450 studies were originally identified, and 4 were included in the final review (see Figure 1 for PRISMA flow diagram). All 4 selected studies [18-21] were RCTs (see Table 1 for study characteristics). Hull *et al.* 2010 [18] was a RCT and topical corticosteroids plus antiviral in the management of RHL was evaluated. A total of 89 patients were recruited. There were cross-overs and there were no losses to follow up. Evans et al. 2002 [19] conducted a trial on 380 participants to evaluate the effect of ME-609 (topical corticosteroid with antiviral) in comparison to placebo. Hull et al. 2009 [20] was a small trial on 39 participants in which valacyclovir and clobetasol gel were compared with placebo. Spruance et al. 2000 [21] conducted a trial on 29 participants where topical corticosteroid with valacyclovir 2gm was compared with antiviral alone. The antiviral used was acyclovir in 2 studies [18,19], famciclovir and valacyclovir in each of the other 2 studies [20,21]. The average number of episodes of the RHL in the participants ranged from 4.5 to 5.6 episodes per year.

**Table 1:** **Characteristics of randomized controlled trials included in the review**

**Study**	**Setting**	**Participants (n)**	**Groups (n)**	**Male, n (%)**	**Frequency of RHL (mean episodes/year)**
Hull et al. 2010 [18]	51 sites in the United States and 4 sites in Canada (July 2006 to December 2007)	1443	Intervention	406 (28)	5.6
			1. ME-609 cream (5% Acyclovir, 1% Hydrocortisone) (n = 601)		
			Control		
			1. Acyclovir (5% in ME-609 vehicle) (n = 610)		
			2. Placebo (vehicle) (n = 232)		
Evans et al. 2002 [19]	4 major university clinics in North America	380	Intervention	76 (20)	5.3
			1. ME-609 cream (5% Acyclovir, 1% Hydrocortisone) (n = 190)		
			Control		
			1. Placebo (n = 190)		
Hull et al. 2009 [20]	University of Utah (August 2004 to March 2007)	39	Intervention	13 (33)	4.5
			1. Oral Valacyclovir (2 g 2×/day for 1 day) plus topical Clobetasol gel (0.05% 2×/day for 3 days) (n = 20)		
			Control		
			1. Placebo (n = 19)		
Spruance et al. 2000 [21]	University of Utah Health Sciences Center, Salt Lake City	29	Intervention	14 (29)	5
			1. Oral Famciclovir (Famvir, 500 mg 3×/day for 5 days) plus topical Fluocinonide (0.05% Lidex Gel 3×/day for 5 days) (n = 17)		
			Control		
			1. Famciclovir and topical vehicle control (n = 12)		

The risk of bias was assessed in the selected studies. The total number of participants included in the studies was 1,892 (range, 29 to 1,443). All four selected studies appropriately reported the process of randomisation. Most of the randomisation achieved 1:1 treatment to control ratios whilst Hull et al. (2010) [18] performed 2.7:1 for the treatment and placebo group respectively [18]. Allocation concealment was also reported in all four selected studies. Therefore, a minimum risk of selection bias was expected in the selected studies as a result of allocation concealment. The participants as well as personnel involved in providing care (medication) were blinded in all four selected studies. All studies reported the use of identical looking substances in both group in similar packing and labels. Two studies clearly reported the blinding of personnel assessing the outcome [18-20]. On the other hand, it was unclear in two studies [19,21]. The intention to treat analysis was used in all four studies and risk of bias was minimal in relation to drop outs and loss to follow up patients’ data.

### Effects of interventions

#### The development of the ulcerative lesions

**Figure 2:**
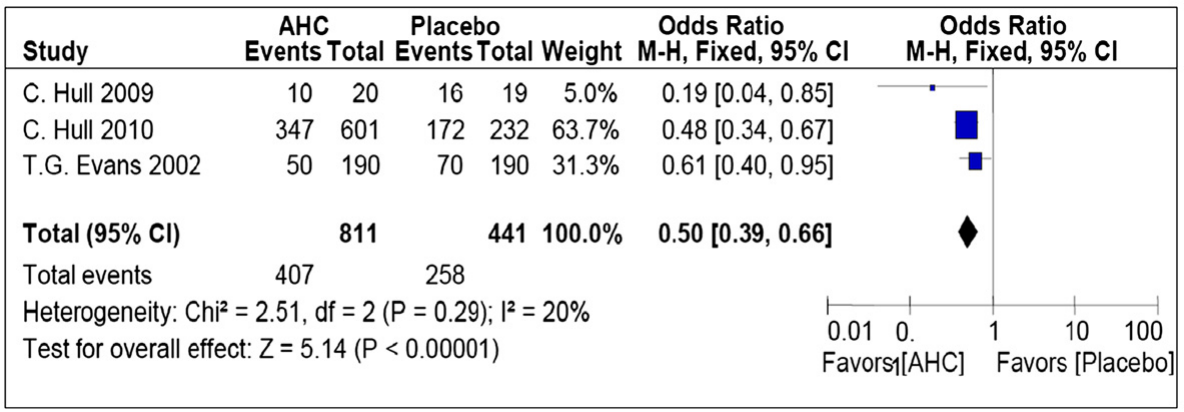
**Forest plot of comparison: topical corticosteroid plus antiviral group versus placebo, outcome: Pooled odds ratio of development of ulcerative lesions.**

Three studies reported on this outcome [18-20]. There was no significant heterogeneity (I^**2**^ = 20%) found amongst trials (Figure 2). The chi-square test for heterogeneity was not significant (P = .29). Meta-analysis showed a statistically significant reduction in the development of ulcerative lesions, demonstrating that the odds of the occurrence of ulcerative lesions were 50% less likely in the intervention group as compared to placebo (OR, 0.50; 95% CI, 0.39-0.66; P < .001).

**Figure 3:**
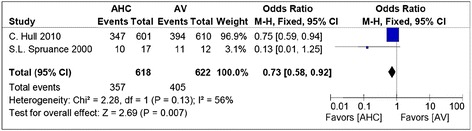
**Forest plot of comparison: topical corticosteroid plus antiviral group versus antiviral alone, outcome: Pooled odds ratio for development of ulcerative lesions.**

Similarly, the comparison of combined therapy with antiviral alone also showed a significant reduction in the development of ulcerative lesions in the intervention arm as compared to the control (Figure 3) (OR, 0.73; 95% CI, 0.58-0.92; P = .007). There was moderate heterogeneity among trials (I^**2**^ = 56%). Chi-square test for heterogeneity was insignificant (P = .13).

#### Healing time for ulcerative lesions (complete epithelisation)

**Figure 4:**
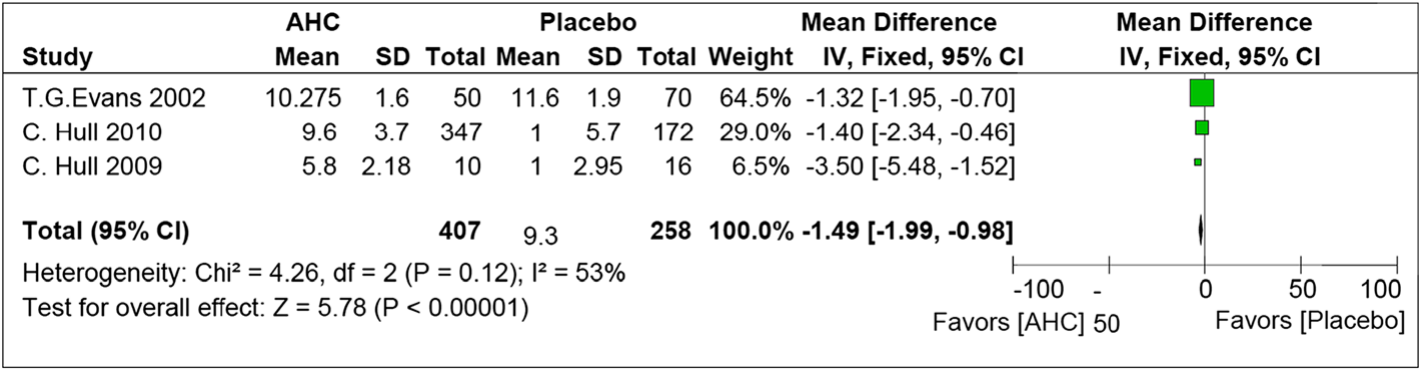
**The comparison of effect of corticosteroid plus antiviral and placebo on the healing time for ulcerative lesions from the first sign until complete epithelisation.**

The meta-analysis of 407 patients showed a significant reduction of 1.49 days in the healing time for ulcerative lesions in the combine therapy group in compared to the placebo group (Figure 4) (95% CI, −1.99 to −0.98; P < .001). This outcome was observed on the based of the healing time from the first sign of lesion until complete epithelisation. All studies showed significant reduction in healing time in the intervention group compared to placebo and there was a moderate heterogeneity between the studies (I^**2**^ = 53%).

**Figure 5:**
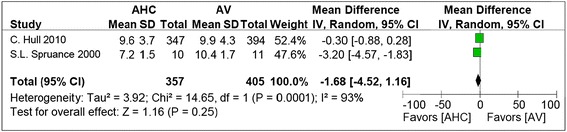
**The comparison of effect of corticosteroid plus antiviral and antiviral alone on the healing time for ulcerative lesions from the first sign until complete epithelisation.**

No significant difference was found in the healing time for ulcerative lesions between the combined therapy and the treatment with antiviral alone (mean difference = −1.68; 95% CI, −4.52 to 1.16) in the pooled data of 357 patients (Figure 5). There was a highly significant heterogeneity between the selected studies (I^**2**^ = 93%), so the random effect model was used.

#### The effect of treatment on pain or tenderness in the lesion

In the comparison of topical corticosteroid plus antiviral with placebo, two of the three studies did not show any significant reduction in pain or tenderness in the intervention group as compared to the placebo. However, the overall effect revealed a significant reduction (OR, 0.59; 95% CI, 0.45-0.77; P < .001). An effect of a smaller magnitude was observed for reduction in pain and tenderness when the combine therapy was compared with antiviral alone. There was a significant heterogeneity (I^2^ = 67%) between the 2 studies in this analysis. The overall effect using the pooled data of 618 participants showed no significant difference in pain between topical corticosteroid plus antiviral group compared to antiviral alone (OR, 0.32; 95%CI, 0.03- 3.76; P = .37).

### Adverse reactions

The most common adverse reactions appeared were dryness, irritation, and stinging symptoms, which were related to topical application and not specifically due to the corticosteroid component. Studies showed no significant difference in proportion of adverse reaction observed between the intervention and the placebo group. There was no heterogeneity between the selected studies (I^2^ = 0%). The overall effect also showed no significant difference between the intervention and the placebo group (OR, 1.09; 95% CI, 0.75-1.59; P = .65).

The odds of the occurrence of adverse reactions were 37% more likely in the antiviral alone as compared to the combine therapy group; however, the effect was not statistically significant (OR, 1.37; 95% CI, 0.97-1.95, P = .08). Heterogeneity between the two groups was not statistically significant (I^**2**^ = 28%).

## Discussion

To date, this is the first meta-analysis to determine the effect of adding topical corticosteroids in the treatment of RHL. The results of this review show that addition of topical corticosteroids to antiviral therapy has significant benefit compared to either antiviral therapy alone or placebo. The review was conducted on RCTs only, which helped in producing rigorous findings about the effectiveness of adding topical corticosteroids in the treatment of RHL.

This review indicates that the chance of developing ulcerative lesions is reduced in patients who receive topical corticosteroids in addition to antiviral treatment in comparison to placebo or antiviral treatment alone. Evidence shows that the treatment with antiviral alone decreases the duration of ulcerative lesions in herpes labialis [22,23]. However, in most cases antiviral treatment alone does not prevent the development of ulcerative lesion [12].

Pain is another important determinant of the morbidity in RHL [6]. A 24% reduction in the development of pain in the lesion was an important outcome observed. One study reported a 26% faster improvement in pain among those patients who received topical penciclovir than those who received a placebo [23]. However, a review of 10 antiviral studies showed that none of them had any significant effect on pain reduction [10]. Furthermore, topical anesthetic treatment alone also did not show any significant impact on pain reduction [24]. Photodynamic therapy is effective in relieving pain in the lesions [25].

The adverse reactions were lower in corticosteroids group than those appeared in the antiviral group alone. A non-randomized clinical trial to determine the safety and tolerance of topical corticosteroids plus antiviral in adolescents found that the treatment was well tolerated and safe [13]. Studies on animals have also shown that the treatment with acyclovir with hydrocortisone is superior to antiviral alone without any significant adverse reactions [26].

The healing time was significantly reduced (around 1.5 days) in the treatment group in comparison to placebo. A review on topical acyclovir revealed that 9 of 13 studies did not show any significant effect on healing time, while 4 studies demonstrated significant reductions ranging from 0.5 to 2 days [27]. Treatment with topical penciclovir alone reduced healing time by 0.5 days in two clinical trials of 3,057 and 1,573 patients [28,29]. Oral antiviral treatment with famciclovir has shown a reduction of approximately 2 days in healing time [30]. Another trial with valacyclovir 2 grams on 1,524 patients found a median reduction in healing time by 1.5 days [31], which is comparable to the reduction in the healing time with topical corticosteroids plus antiviral demonstrated in this review.

There are a number of limitations of this study. First, the meta-analysis in this study was conducted on a small number of studies, which is likely attributable to the limited availability of topical corticosteroid plus antiviral treatment. The statistical tests to identify publication bias could not be performed due to the small number of studies in the review. Second, the selected studies used different form and type of antivirals (oral and topical). The topical antiviral agents that are most commonly recommended to treat RHL include acyclovir 5% cream, penciclovir 1% cream and docosanol 10% cream. Most of these preparations need to be applied every 2 hours from the time of prodrome until complete healing [32]. Unlike topical agents, systemic medications enable greater drug exposure, rapid access to site of viral replication, better biocompatibility, less frequent dosing, and improved compliance. Systemic antiviral agents may be administered orally or intravenously. Acyclovir has a short half-life and multiple doses are required to maintain an optimum drug levels in serum. Famciclovir and valacyclovir have greater bioavailability and are more convenient for patients [33]. The reduction in healing time for oral antiviral treatment is generally higher than topical antivirals; this may have had an impact on the outcomes of this study. Finally, 2 studies used ultraviolet-induced cold sores, which may lead to more severe outbreaks. Ultraviolet light is known to be a stimulus for the reactivation of herpes simplex virus. Ultraviolet-induced cold sores may develop very rapidly [34,35]. However, in this study, we found that the combination therapy was equally effective for the ultraviolet-induced cold sores.

## Conclusion

The addition of the steroidal component along with antivirals improves treatment of RHL. Adverse reactions are lower in topical corticosteroid plus antivirals treatment than treatment with antiviral alone. Thus, the treatment with topical corticosteroid plus antiviral alone is safe and more effective in preventing the development of ulcerative lesions than antiviral alone or placebo. The healing time may not be further reduced with topical corticosteroid plus antiviral than with antiviral treatment alone.
